# A record of igneous evolution in Elysium, a major martian volcanic province

**DOI:** 10.1038/srep43177

**Published:** 2017-02-24

**Authors:** David Susko, Suniti Karunatillake, Gayantha Kodikara, J. R. Skok, James Wray, Jennifer Heldmann, Agnes Cousin, Taylor Judice

**Affiliations:** 1 Department of Geology and Geophysics, Louisiana State University, Louisiana, USA; 2 Department of Oceanography and Marine Geology, Faculty of Fisheries and Marine Sciences & Technology, University of Rahuna, Matara, Sri Lanka; 3 SETI Institute, California, USA; 4 School of Earth and Atmospheric Sciences, Georgia Institute of Technology, Atlanta, GA, USA; 5 NASA Ames, California, USA; 6 Institut de Recherche en Astrophysique et Planétologie, Toulouse, France

## Abstract

A major knowledge gap exists on how eruptive compositions of a single martian volcanic province change over time. Here we seek to fill that gap by assessing the compositional evolution of Elysium, a major martian volcanic province. A unique geochemical signature overlaps with the southeastern flows of this volcano, which provides the context for this study of variability of martian magmatism. The southeastern lava fields of Elysium Planitia show distinct chemistry in the shallow subsurface (down to several decimeters) relative to the rest of the martian mid-to-low latitudes (average crust) and flows in northwest Elysium. By impact crater counting chronology we estimated the age of the southeastern province to be 0.85 ± 0.08 Ga younger than the northwestern fields. This study of the geochemical and temporal differences between the NW and SE Elysium lava fields is the first to demonstrate compositional variation within a single volcanic province on Mars. We interpret the geochemical and temporal differences between the SE and NW lava fields to be consistent with primary magmatic processes, such as mantle heterogeneity or change in depth of melt formation within the martian mantle due to crustal loading.

The lava fields surrounding Elysium Mons and its neighboring super-shield volcanoes, Albor Tholus and Hecates Tholus (Fig. 1), record some of the most recent volcanism on Mars^1^,^2^,^3^,^4^. Most of the surface of Elysium Volcanic Province dates to the Amazonian period^5^,^6^,^7^ (beginning between 3.3 and 2.9 Ga), with a considerable areal extent of Late Amazonian age (beginning 0.6 to 0.3 Ga)^4^. Past work estimates that the age of the basement lava flows, the stratigraphically lowest and oldest we are able to observe in the region, date to ~4 Ga^4^. Other studies have identified late periods of volcanic activity in isolated areas of the southern portion of the Elysium Volcanic Province to extend to the last 250 Ma, including some remarkably young episodes as recent as 16.2–13.5 Ma, 4.3 Ma, and 3–2.5 Ma^8^. These dates highlight The Elysium Volcanic Province as particularly interesting, given its long and continuous history of volcanic activity. This long history suggests that these lavas could record varied magmatic evolution that can provide important constraints on the evolution of Mars as a geologically active and diverse planet. The Elysium Volcanic Province may also provide important insight for the geologic evolution of Amazonian terrains, given its geographic isolation in the northern lowlands away from other volcano-tectonic regions.

Mid-latitude (exclude latitudes poleward of ~±45°) mapping of mass fraction distributions of the 9 elements: Al, Ca, Cl, Fe, H, K, Si, S, and Th, obtained from the γ spectral data from the Mars Odyssey Gamma and Neutron Spectrometer (GRS) instrument suite^2^,^9^,^10^,^11^,^12^,^13^, showed a unique geochemical signature for Amazonian lava flows^7^ in the southeastern portion of the Elysium volcanic province (Fig. 1). Here, work by Karunatillake *et al*.^2^ identified depletions in both K and Th (two elements characterized by the strongest geochemical affinity during igneous processes)^9^,^14^ by more than the combined standard deviation and typical standard error relative to their respective global averages. The depletion in these elements indicate that the southeastern lava fields are chemically anomalous when compared to the compositions of the martian mid-latitudinal regolith to decimeter depth scales^11^. These anomalous geochemical signatures identify the Southeast Elysium lava fields as promising candidates for further geochemical investigation.

Recently, Baratoux *et al*.^3^ used γ data from the Northwest Elysium lava fields (Fig. 1) to investigate the thermal history of Mars during the Amazonian period. These authors investigated the composition of the lavas, and compared them to the compositions of Hesperian and Amazonian volcanic provinces elsewhere on Mars^3^. Abundances of SiO_2_, FeO and ThO were used to estimate mantle potential temperature, degree of partial melting, and lithospheric thickness in correspondence with these volcanic provinces when these eruptions took place^3^. In this work, we compare the Southeast (SE) Elysium region to the Northwest (NW) Elysium region in order to constrain the spatial and temporal variation of composition of lava flows across a single martian volcanic province.

Previous investigations in the Radar Stealth region^2^ suggests that data from the High Resolution Imaging Science Experiment (HiRISE), on board the Mars Reconnaissance Orbiter (MRO), can lead to inferences about the physical properties of surface-to-subsurface material. In this work we use HiRISE imagery to complement in our interpretation of geochemical signatures.

Cerberus Fossae, an area overlapping the southern portion of the chemically anomalous region in SE Elysium, contains evidence of chemical layering involving volatile elements in the subsurface, potentially due to complex interactions among lava flows, volcanic aerosols and eolian sediment^15^. Morphologies, such as mesas and other related features in close proximity to Cerberus Fossae, have been ascribed to phreatomagmatism^1^,^16^,^17^, lava-water interactions^18^ and relict ice floes^19^,^20^. Observations of patterned ground with a remarkable similarity to terrestrial periglacial environments^21^ also suggest the likelihood of geologically recent aqueous processes. All these processes could explain some of the observed chemistry. However, radar sounding data appear consistent with interbedded lava flows and weakly compacted sediments or porous rocks^22^.

Pure water is unstable on the surface of Mars^23^,^24^,^25^. Brines, however, could have been free-flowing on the surface and could still be present in subsurface reservoirs of Mars^26^, and thus could be thought of as agents for alteration of surface rock. Common secondary minerals on Mars, such as Ca- and Mg- Sulfates and Fe-Oxides could be deposited by low pH, sulfur-rich, aqueous solutions in very low water-to-rock ratio, and subsequently, limited Al-mobilization conditions^27^. Evaporite minerals, such as Halite (NaCl) and anhydrite (CaSO_4_), could have precipitated following evaporation of highly saline aqueous solutions. If the volatiles detectable by the GRS, (S, Cl, and H_2_O) are enriched in either SE or NW Elysium, this could support the presence of such minerals at regional scales.

In addition to observations made by orbiting spacecraft, we make comparisons between the regional chemistry in Elysium and *in situ* samples analyzed from rover landing sites on Mars and the martian meteorites. The compositional data for several martian meteorites as well as *in situ* samples from Gusev Crater, analyzed using the Alpha Particle X-ray Spectrometer (APXS) onboard the MER Spirit Rover, are used for comparison with the γ data. The martian meteorites, which have been used in the past to model the compositions for bulk silicate Mars^28^,^29^, provide the most detailed information about the martian mantle’s evolution and magmatic differentiation of any data set available to us. In order to better understand igneous alteration trends, which may be applicable to the Elysium Volcanic Province, we use previously categorized *in situ* rock samples from Gusev, which have been divided into unaltered and altered igneous samples^30^,^31^,^32^,^33^. In this study, we were able to rule out the presence of a secondary geochemical signature, and establish igneous processes as the principal factor controlling Elysium’s anomalous compositions.

## Geologic Setting and Characteristic Geomorphology

**Figure 1:**
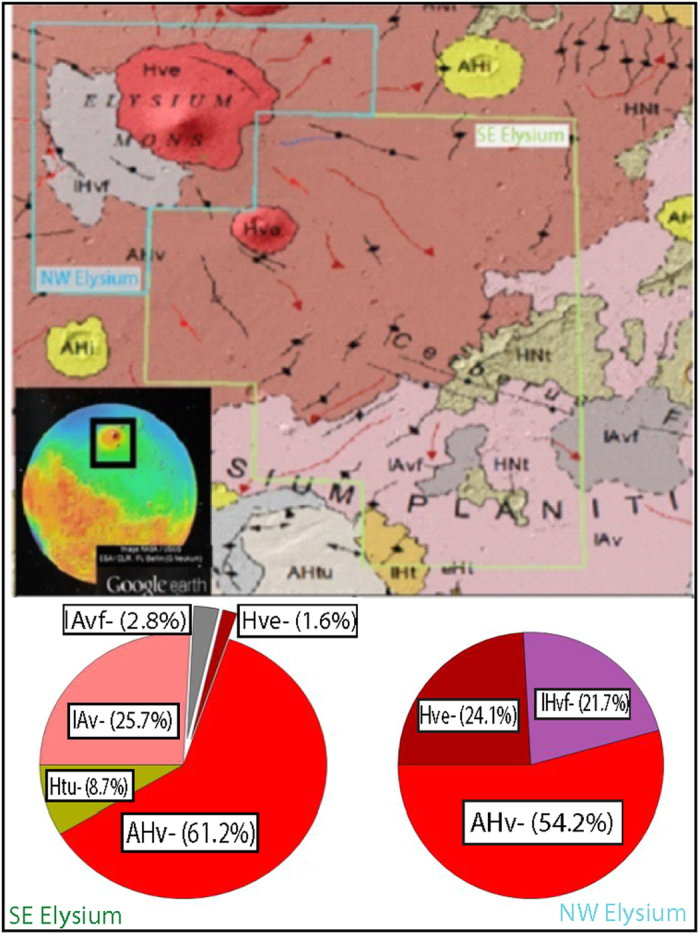
Location and geological setting of the Southeast (Green) and Northwest (Blue) Elysium lava fields with corresponding GRS pixels. Geology map adopted from the work by Tanaka *et al*.^6^ and made publically available at http://pubs.usgs.gov/sim/3292/pdf/sim3292_map.pdf. Inset image is a global reference map for Mars with overlain MOLA topography from Google Earth. The areal fraction of each unit within each region is quantified using ArcGIS software (pie charts). AHv is”Amazonian and Hesperian Volcanic unit”, lAv is “Late Amazonian Volcanic unit”, lAvf is “Late Amazonian Volcanic field unit”, lHvf is “Late Hesperian Volcanic Field unit”, Hve is “Hesperian volcanic Edifice unit”, Htu in the pie charts stands for “Hesperian Transition units” and is a combination of 3 geologic units: HNt (Hesperian-Noachian transition unit), lHt (Late Hesperian transition unit), and AHtu (Amazonian-Hesperian transition unit).

**Figure 2:**
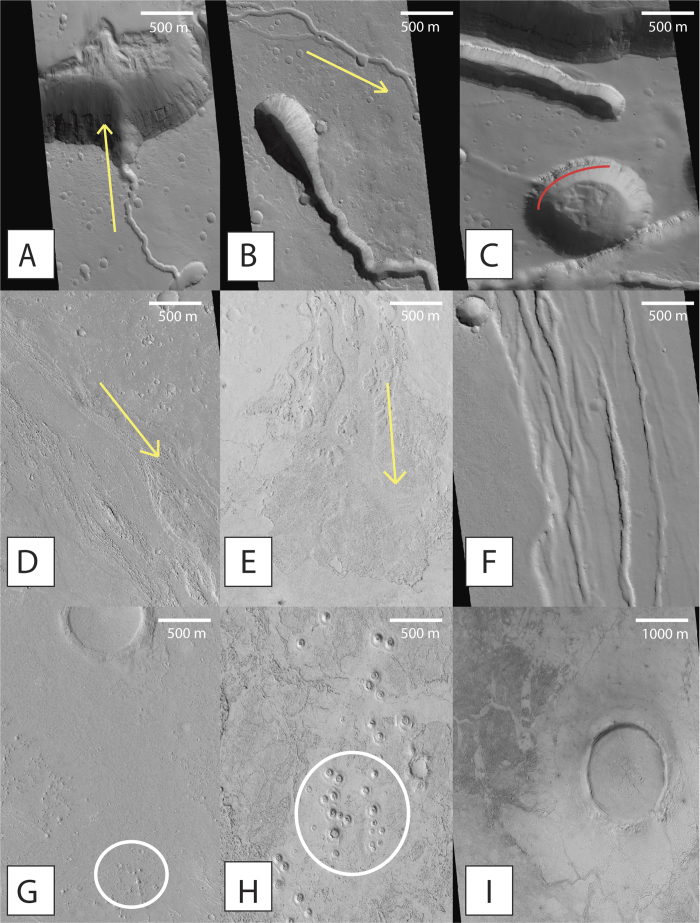
Selected HiRISE imagery from both SE and NW Elysium regions. (**A**) ESP_022915_2070 (26.949°N, 142.844°E) shows flows down a crater slope in NW Elysium. (**B**) ESP_013157_2015 (21.498°N, 149.738°E) shows lava flows on the south flank of Elysium Mons within NW Elysium. (**C**) PSP_004046_2080 (27.49°N, 143.21°E) shows fissures and pit crater collapse feature of lava tubes in the NW region. The collapse of the lava tube has exposed stratified lava flows. (**D**) ESP_037802_1880 (8.1°N, 154.7°E) shows lava channels and accompanying lava levees within SE Elysium. (**E**) PSP_005984_1850 (5°N, 156.4°E) shows pa’hoe’hoe style flows and a lava-draped channel at its terminus within SE Elysium. (**F**) ESP_014278_2050 (24.71°N, 143.6°E) shows the lava flow boundary between separate geologic units HVe and lHvf in the NW region. (**G**) ESP_028466_1955 (15.22°N, 162.45°E) shows an image from the SE with an infilled crater in the top half of the image and rootless cones (circled) in the bottom half. (**H**) ESP_012524_1855 (5.58°N, 153.07°E) shows more prominent rootless cone structures as well as abundant Pa’hoe’hoe analog style flows within SE Elysium. (**I**) ESP_034967_1885 (8.48°N, 149.19°E) shows a large crater infilled by the lava flows from SE Elysium. North is up in all pictures. The yellow arrows represent inferred direction of flows.

The Elysium lava flows erupted from the edifices of the three super-shields, from volcanic fissures, and from at least 22 topographically low shield volcanoes scattered across the Elysium Volcanic Province^8^. Using the most recent mapped geologic units of Mars^6^,^7^, we quantified the areal fraction of each geologic unit comprising both NW and SE Elysium (Fig. 1). The SE Elysium region (1.37 × 10^6^ km^2^ in areal extent) is dominated by Amazonian volcanic units, which comprise nearly 90% (1.23 × 10^6^ km^2^) of its areal fraction. The most abundant unit type is the Amazonian-Hesperian volcanic unit (AHv), making up more than 60% (8.22 × 10^5^ km^2^) of the surface (Fig. 1). The AHv contains low viscosity flood lavas and stacked, gently sloping, lobate flows of highly variable ages, which are sourced from vent systems and local fissures rather than large volcanic edifices^6^. Late Amazonian volcanic units and volcanic fields, lAv and lAvf respectively, make up nearly 30% (4.11 × 10^5^ km^2^). They contain planar deposits consisting of troughs, ridges, and platy textures which we identified in HiRISE image Fig. 2F. These units are young, and include pristine flows and occasional sinuous lava channels (Fig. 2D). The rest of the region is made up of Hesperian transition units (Htu) and the Hesperian Volcanic Edifice (HVe) of the super-shield Albor Tholus^6^ (Fig. 1).

Several Hesperian geologic units are mapped in the NW region of Elysium (5.06 × 10^5^ km^2^ in areal extent)^6^,^7^. While the majority of the region is characterized as AHv (~54% of the surface, or 2.72 × 10^5^ km^2^), a significant portion (~22% or 1.11 × 10^5^ km^2^) of the surface is covered by the Late Hesperian volcanic field unit (lHvf). The lHvf unit is hundreds of meters thick and is made up of far flowing, low viscosity lavas which are locally modified by troughs and fissures^6^, such as those shown in Fig. 2C. The remaining ~24% of the NW region (1.21 × 10^5^ km^2^) is covered by the HVe unit (~24%), which makes up the edifice of the super-shield volcano Elysium Mons^6^. These Hesperian units, which did not experience the same Amazonian volcanic resurfacing events as SE Elysium, contribute substantially to the observed geochemistry in the NW region.

Morphologies indicative of effusive volcanic eruptions are observed throughout both the SE and NW Elysium regions. Figure 2 shows some representative morphologies. In terrestrial settings, basaltic magma often generates effusive eruptions that build deep, sinuous lava channels^5^, which are analogues to features shown in various HiRISE images. Terrestrial observations, suggesting greater flow distance with lower viscosities remain applicable to the Elysium lava flows (Fig. 2A). These images also show other low viscosity morphologies such as infilled craters, lobate flows, and platy ridge flows. Pa’hoe’hoe lava features form when low viscosity lava cools and becomes more viscous, this results in an over-folding, ropy morphology. Analogous features are present within the SE Elysium region (Fig. 2E and H). The crater shown in Fig. 2C, located in NW Elysium, is not an impact crater, but is thought to be a collapse feature of a lava tube, as evident by the presence of scalloped walls and the lack of ejecta blanket, raised rim, or central peak^1^. This collapse feature exposes layered lava flows, effectively revealing the stratigraphic record of lava flows that have created the Elysium Volcanic Province over its geologic history^4^,^8^.

Other morphologies may suggest the presence of alternative geologic processes taking place concurrently with these lava flows. Figure 2H shows rootless cone structures, identifiable based on their clustered behavior on top of lava flows and the lack of extruded material around their circular bases^18^. These features have been interpreted to be a product of lava flow emplacement over a hydrated surface^16^. The local ground water or ice is volatilized by the hot, flowing, lavas and creates a phreatomagmatic explosion resulting in these cones^16^,^18^,^34^,^35^. These features, also visible roughly 1 km south of the infilled crater in Fig. 2G, were identified in the SE Elysium region, but were not observed in the NW.

## Results

**Figure 3:**
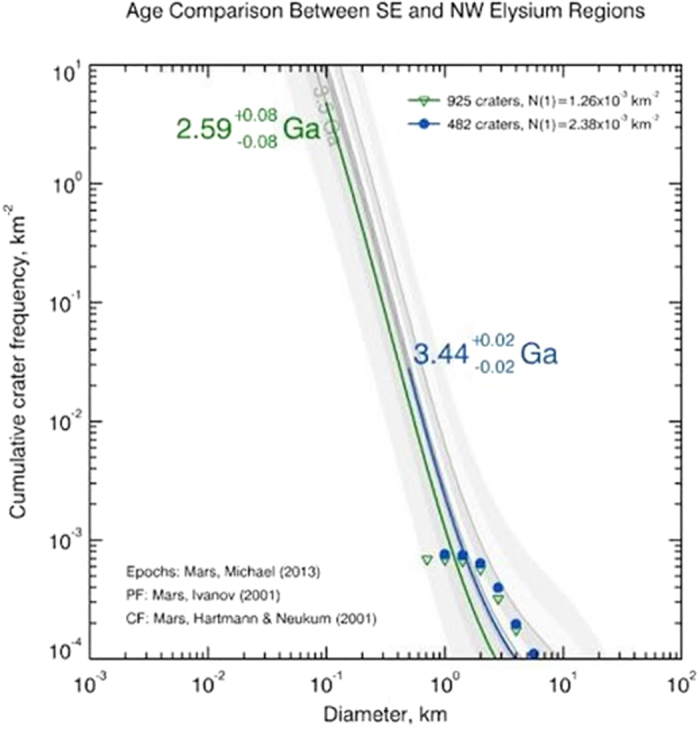
CraterStats2 results for planetary surface age dating using crater counting^7^,^55^. 925 craters greater than 1 km in diameter were identified for the SE Elysium region, while only 482 craters of the same size were identified in the smaller NW region. This figure plots Diameter of Craters vs Cumulative Crater Frequency. Southeast Elysium (green) is estimated to be younger at 2.59 ± 0.08 Ga and Northwest Elysium (blue) older at 3.44 ± 0.02 Ga.

While past analysis has looked at the ages of Elysium Planitia broadly^4^ or on a more localized basis^8^,^35^, we sought to estimate the average age of all the lava flows within surface areas of similar regional geochemistry within Elysium^2^,^3^. The total number of craters (N) greater than 1 km in diameter was found to be N = 925 for the SE Elysium region and N = 482 for the NW. The cumulative crater frequency reveal the average age of the surface in SE Elysium province is 2.59 ± 0.08 Ga, while the surface in NW Elysium is estimated to be 3.44 ± 0.02 Ga (Fig. 3). Both dates are comparable to previous studies of the ages of various lava flows within Elysium, which estimated the oldest lava flows to range between 4 and 2 Ga^4^.

Ratio bound box plots^2^ (Methods) show meaningful differences in compositional distributions between the SE and NW regions. In this work, an elemental abundance in a region “A” is refered to as a “major” enrichment or depletion, relative to a region “B”, when the lower (25^th^%tile/75^th^%tile) or upper (75^th^%tile/25^th^%tile) bound, respectively, surpasses unity. In turn, the enrichment or depletion is considered “minor” when the ratio of medians surpasses unity but the lower or upper bounds do not. An abundance is considered similar between the two regions if the ratio of the 50^th^%tile/50^th^%tile is within one standard error. We list the error-bound median ratio in parenthesis following the element.

**Figure 4:**
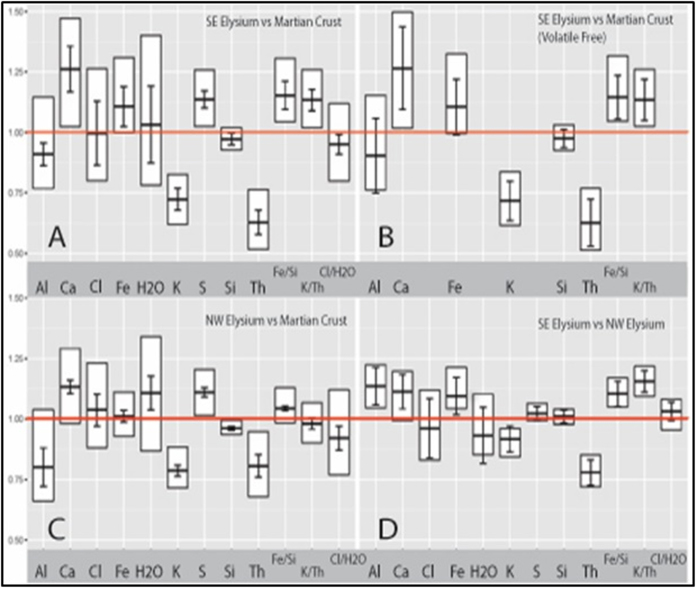
Ratio bound box plots indicate compositional ratios of one target region to another as 75^th^/25^th^ percentile, 50^th^/50^th^ percentile, and 25^th^/75^th^ percentile for the mass fraction of the elements: Al, Ca, Cl, Fe, H2O, K, S, Si, Th, and the ratios of Fe/Si, K/Th, and Cl/H2O. Deviation of the median ratio from unity would suggest tentative compositional distinctness, while the lack of overlap with unity for a given box would show a significant change, even for small relative differences. Error bars were calculated as the uncertainty of the median average deviations (MAD) using the equation MAD = median [|X_i_-median (X_i_)|], where median (*X*_*i*_) is the median of all values X_i_^54^. The MAD of a set of values is particularly robust and is more resilient against outliers in the data when compared to the standard deviation. Red lines highlight the 1 to 1 ratio, values below the line represent depletion, while values above represent enrichments. (**A**) Chemical differences between the Southeast Elysium region and the chemical average for the martian crust. (**B**) SE Elysium vs martian crust, normalized for an observed volatile-free basis, removing Cl, S, and H2O from the bulk composition. (**C**) NW Elysium Chemical province vs martian crust. This plot highlights the similarities in trends away from the martian crust compositions for both the NW and the SE regions (shown in a). (**D**) NW Elysium Chemical province vs the SE Elysium chemical province, showing the differences between regions, most notably in Ca, Fe, K, and Th.

The first ratio bound box plot compares the regional geochemistry of the SE Elysium region to the composition of the average martian crust (Fig. 4A). In addition to the previously identified major depletions of K (0.72 ± 0.05) and Th (0.63 ± 0.05)^2^, this region differs significantly from the typical martian crust in several elements detectable with γ spectra, as shown by Fig. 4A. The region has a minor depletion in Si (0.97 ± 0.02) and Al (0.91 ± 0.05), while Ca (1.26 ± 0.10) and Fe (1.11 ± 0.08) display major enrichments throughout the region. For example, the lowest values, represented in the 25^th^ percentile, of Ca and Fe, exceed most of the highest values, represented in the 75^th^ percentile, of the martian crust. This amounts to enrichments in Fe to over 16% of the bulk composition in some parts of the region, which is significant, considering that average for the martian crust is only 12.6 ± 0.7%. A major regional enrichment in S (1.14 ± 0.04) is also observed.

Mars has a history of global-scale dust storms which mobilize layers of high albedo fine material of few microns to tens of micron size scale (i.e., dust) across the planetary surface^36^. Elysium in particular has a thick layer of this dust^37^. We renormalize the γ derived geochemistry to a volatile and “mobile” element-free basis in order to ensure the compositions investigated in Elysium represent the igneous components at the surface, rather than those affected by interference from the dust. The normalization factors were calculated for each pixel using the standard equation 100/(100-[H_2_O]-[SO_3_]-[Cl])^10^. A constant oxidation state of +6 is assumed for S when converting to SO_3_^10^. The results (Fig. 4B) reveal that the volatile-free compositions show the same relative trends between the SE and the martian crust as the volatile-bearing compositions, indicating the compositions are cation-conserving^27^, and volatiles merely dilute the abundances of the major elements, rather than dominate a surficial layer.

Compared with the average martian crust, the NW Elysium region has minor enrichments in Ca (1.13 ± 0.03) and H_2_O (1.11 ± , 0.07), as well as a major depletion in Si (0.96 ± 0.01) and a major enrichment of S (1.11 ± 0.02). The NW region also shows a minor depletion in Al (0.80 ± 0.08) and major depletions in K (0.79 ± 0.02) and Th (0.81 ± 0.05). These results are shown in ratio bound box plot for Fig. 4C.

When compared to one another, the two regions of the Elysium Volcanic Province reveal several interesting geochemical trends (Fig. 4D). The SE Elysium region displays a minor enrichment of Ca (1.11 ± 0.07) and major enrichments in Al (1.34 ± 0.08) and Fe (1.09 ± 0.08). Both regions have nearly identical abundances of Si (1.02 ± 0.03). Despite both regions having major depletion in the high spectral precision elements, K and Th, relative to the martian crust, the depletion is even more significant in the SE region, which shows a major depletion relative to the NW.

**Figure 5:**
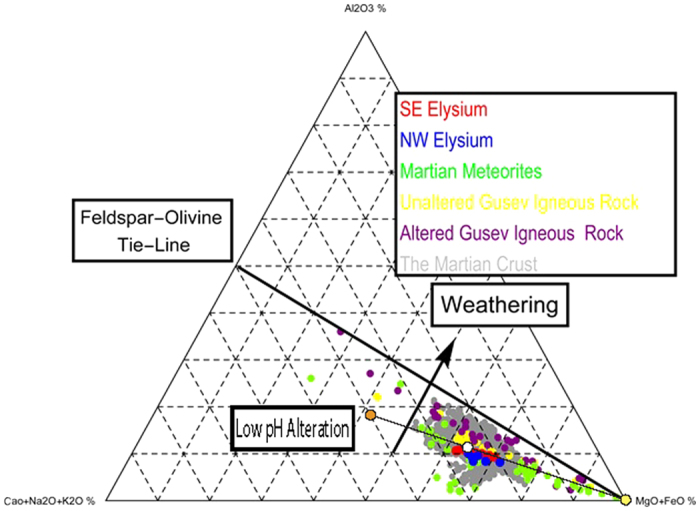
Ternary plot of (Al_2_O_3_) − (CaO + Na_2_O + K_2_O) − (MgO + FeO) as a proxy for the index of alteration for martian rocks. The black ellipse represents the range of values for the martian crust as detected by GRS. Open system aqueous alteration trend is shown with the black line, with altered rock (left circle), unaltered rock (middle), and alteration fluid (right). This line corresponds to the results from the alteration of synthetic martian rocks under low pH laboratory conditions by Hurowitz and McLennan^29^. Plots the pixels from both SE (red) and NW (blue) Elysium vs the martian meteorites (green), unaltered Gusev igneous rock (yellow), altered Gusev igneous rock (purple) and the rest of the GRS data (gray). The data sets form a trend in the direction of more exposure to alteration effects, with the martian meteorites representing compositions of little-to-no alteration, and the altered Gusev samples representing the most heavily altered igneous compositions.

We use molar ternary plots^27^,^31^,^33^,^38^ to further our geochemical analysis of the Elysium provinces and test whether aqueous alteration could explain the observed geochemical anomalies. We selected a ternary diagram which has been used to describe the aqueous weathering trends of olivine‐bearing basalts, given their pervasiveness in the martian crust, under both moderate pH regimes of typical terrestrial settings^38^ and low pH conditions, with low water to rock ratios, where most chemical weathering processes on Mars occur^27^. The apices of this ternary are (Al_2_O_3_) − (CaO + Na_2_O + K_2_O) – (MgO + FeO) [A-CNK-MF] (Fig. 5). For the moderate pH conditions on Earth, altered rocks progress from below the feldspar-olivine tie-line, which connects the pure end-member compositions of general feldspar [(K,Na,Ca)(Al,Si)_2_Si_2_O_8_] and olivine [(Mg,Fe)_2_SiO_4_], toward the Al_2_O_3_ composition^38^,^39^. This terrestrial trend is shown by the black “weathering” arrow in Fig. 5. The second trend-line corresponds to the results from the alteration of synthetic martian rocks under low pH laboratory conditions, with the altered rock progressing toward to A-CNK edge of the ternary and the alteration fluid progressing toward to MF apex^27^. Even though the ternary plots have both Na_2_O and MgO components, these oxides lack the associated γ-derived chemical data. Consequently, we use the method by Baratoux *et al*.^40^ to estimate Na_2_O and MgO mole fractions (Methods).

Figure 5 shows the majority of the Elysium chemical provinces’ pixels plotting directly on the low pH system aqueous alteration trend line^32^. Pixels from both the NW region (blue) and the SE region (red) overlap and plot near the alteration under low pH trend line (dashed line), close to the unaltered rock composition (white disk), away from the altered composition of basalts (orange disk). The martian meteorites, generally plot below the trend line for low pH aqueous alteration, closer to the CKN-MF edge of the ternary. The APXS-analyzed *in situ* rocks from Gusev Crater generally plot between the low pH aqueous alteration trend line and the Feldspar-Olivine Tie-Line. The unaltered igneous samples plot closer to the martian meteorites, while the altered rocks plot above them, closer to the feldspar-olivine tie-line. The NW and SE Elysium pixels plot on the trend line between the martian meteorites and the unaltered Gusev igneous rock.

## Discussion

In this work, we identify a compositional shift temporally and spatially across the Elysium Volcanic Province from the older NW region (3.44 ± 0.02 Ga) to the more recent SE region (2.59 ± 0.08 Ga). Through the convergence of multiple data sets, we quantify the compositional changes in lavas of variable ages across the province. Our results indicate that the unique geochemistry of the Elysium Volcanic Province represents igneous processes as opposed to secondary aqueous alteration of the surface or interference from eolian superficial dust.

The resulting 0.85 ± .08 Ga difference between the SE and NW Elysium regions estimated by crater counting provides the province with a record of varied magmatic compositions over its geologic history. This in turn, helps to constrain the evolution of Mars as a geologically active planet. The Hesperian/Amazonian temporal boundary is estimated to be between 3.3 to 2.9 Ga, but could also vary as much as from 3.4 to 2.0 Ga due to poorly constrained martian cratering and erosional rates during the time^41^. With this chronology in mind, the SE Elysium region is decidedly Amazonian, while the older NW Elysium province falls at end of the late Hesperian era, just prior to the Hesperian/Amazonian Boundary. Consequently, prior analyses by Baratoux *et al*.^3^ that focused exclusively on the NW region, may not represent the thermal history of the Elysium Volcanic Province during the Amazonian period as directly as the SE province.

The NW region is depleted in Al, Ca, and Fe compared to the SE region, while K and Th show significant depletions in the younger flows. In order to compensate for the observed depletions in Al, Ca, and Fe, an unreported major (>10%) rock-forming element must be enriched in the NW to sum the total oxide mass fractions detected by the GRS up to unity for mass balance purposes. The only major rock-forming element not reported by the GRS is Mg, making it a reasonable candidate for the majority of the missing mass fraction. These differences in major element abundances between the two regions have major implications for time dependent changes to the composition of the martian mantle from the late Hesperian to the Amazonian periods.

Elemental ratios can be used to assess the likelihood of secondary geologic processes. Elysium, like much of the northern mid-latitudes, has a surficial layer of fine material dominated by tens of microns grain size, or “dust”^42^,^43^. This dust is characteristically bright, having high albedo and low thermal inertia, and has been found to partially dominate the geochemical signature of large regions of the planet^44^. Bright dust at the MER rover sites, Gusev and Meridiani, was analyzed using the APXS instrument and found to contain elevated abundances of the more mobile and volatile elements, S and Cl, relative to darker, less mobile soil and rock^45^. Evidence of abundant hydrated minerals within the soils was also found using the MER rovers’ MiniTES instrument^46^. An abundance of dust could cause the elevated levels of S in the SE and both S and H_2_O in the NW^46^,^47^. If the primary source of volatile elements at Elysium is attributed to a layer of globally-sampled dust deposited by atmospheric processes, then the chemical province should yield a Cl/H_2_O ratio similar to the global average. Rather, we observe a lower Cl/H_2_O ratio in both the SE and NW regions relative to the martian crust (Fig. 4A and C). More likely, the distinct Cl/H_2_O ratio and enrichment in S reflect the volatile content of the magma which formed the Elysium Volcanic Province, as these volatiles are readily sourced from volcanic degassing^12^.

Past work has described dust within the most heavily mantled regions of Mars to be enriched in K and Th rather than depleted^44^. These heavily mantled regions, which include Arabia, Tharsis, and Amazonis, are chemically distinct from one another, indicating that the γ signature is reflective of locally, rather than globally, derived material^11^,^44^. If this is the case in Elysium, even if the GRS is sampling the signature from the dust, it still reflects the igneous compositions of the region. If the γ signature had been influenced by the sampling of a thick layer of volatile-enriched dust on top of the bulk basaltic crust, the renormalizing to a volatile-free basis would have caused an apparent enrichment in the remaining elements. The volatile-free ratio bound box plot (Fig. 4B) comparing SE Elysium to the martian crust does not reveal any deviation from the relative abundance trends compared to the bulk compositions. This supports the idea that the GRS is primarily sampling the bulk basaltic rock composition.

Effusive lava flows, which result from hot magma outpouring onto the surface, can be greatly influenced by H_2_O in its source; even just a few ppm of water in the lava could greatly reduce its viscosity^5^, and NW Elysium shows a minor enrichment of H_2_O in the γ data relative to the martian crust. As mentioned previously in this work, morphologies characteristic of effusive flows such as ropey, lobate textures, sinuous channels, and lava tubes dominate the landscape across both regions of Elysium. The rootless cone structures within SE Elysium could indicate a wet environment with higher than average water-to-rock ratios on the surface during the time of emplacement of these lavas, but they do not appear to be widespread enough to dominate regional geochemistry. In addition to morphology, calculations done by prior work for the temperature of NW Elysium magmas also favor a relatively hot source, with mantle potential temperatures of 1405 °C, the highest of any Amazonian volcanic province analyzed by that work^3^. This high temperature would suggest a deep mantle origin and would agree with the undepleted magma compositions with high abundances of volatiles, such as H_2_O, that are observed in the γ data.

The temporal variation between the two regions is consistent with magmatic variability and provides a degree of insight into the igneous evolution of the Elysium Volcanic Province. The observed geochemical trends suggest a difference in source signature between the older NW region and younger SE region (Fig. 4). Two possible interpretations arise to explain these temporal variations in geochemistry. The first is that of a heterogenous mantle, with pockets of distinct compositions contributing to the geochemical signatures we observe on the surface at different periods in martian history. Multiple models exist to describe how the martian mantle might be compositionally heterogeneous both laterally and vertically. The possibility of a whole-mantle magma ocean on early Mars has been speculated. The model predicts solidification of multiple zones of cumulates in the mantle at different depths, which eventually differentiate into distinct lateral components when the deeper cumulates rises and settle at neutral buoyant forces nearer to the shallow zone^48^. This mantle overturn leads to lateral heterogeneity, with multiple zones of cumulates of different densities, within mantle sources. Differences in mineral content is expected to be observed in the eruptions from these sources, with regions of dominated by specific minerals, with lower density cumulates being enriched in MgO and higher density cumulates being enriched in FeO^48^. It is worth noting that Fe, which other than Si, is the most abundant element detectable by the GRS and shows significant variability between the two regions. Fe may prove to be a more useful indicator of mantle overturn at regional scales across Mars.

Recent work by Balta & McSween^49^ has described a separate model for martian mantle evolution which could lead to source heterogeneity. This model proposes that older Hesperian volcanoes were sourced from a portion of the upper depleted mantle, while the younger Amazonian volcanoes were occasionally sourced from upwelling from a deeper undepleted portion of the mantle. This upwelling provides the surface with water-rich, shergottite-like compositions^49^. These lavas of distinct age and composition within Elysium could potentially represent a regional expression of this global mantle evolution. This is unlikely, however, because the compositions observed in the two regions of Elysium (i.e. relative enrichment in Fe and H_2_O for the younger SE region) do not match expectations of the model by Balta & McSween^49^, which predicts higher concentrations of Th and H_2_O and depletions in Fe for the younger eruptions.

The second interpretation of possible igneous processes exists as a change in depth of the melt formation in the mantle beneath the Elysium Volcanic Province. This is made possible by a model which predicts continual mantle plumes rising to the surface throughout Elysium’s lengthy eruptive history during the late Hesperian and Amazonian Eras^1^. The loading of the lithosphere by the formation of the super-shields could cause an increase in pressure on the mantle source over time, depressing the geothermal gradient. This increase in pressure would be accompanied by a decrease in degree of partial melting and an increasing depth of melt formation. The younger volcanism in the SE, where lithospheric thickness is considerably lower^40^, would take place with lower pressures and at shallower depths. With distinct depth to melt formations between the Hesperian and Amazonian, magmas erupted with notably different abundances of elements. This is supported by the compositional distinctness between the NW region and SE region of several major and minor elements (Fig. 4D), most notably Th and Fe. The higher concentration of Th in NW Elysium suggests a lower degree of partial melting in the mantle source^3^, while the elevated Fe abundance in the SE is likely indicative of a change to a shallower source depth^50^ in SE Elysium.

Based on our results, the geochemistry seems most consistent with a model for the change in the depth of melt formation in the mantle beneath the Elysium Volcanic Province. This conclusion has major implications for the history of martian mantle evolution, such as how volcanic provinces were built up over time, and motivates future investigations into this province. Petrologic modeling techniques using pMELTS and SPICEs programs on the bulk silicate compositions of Mars proposed by Taylor^29^ might help us to constrain how the compositions of volcanic provinces changes with variable pressures and degrees of partial melting.

## Methods

In order to investigate a potential sub-era variation in volcanism, we use mapped geologic units in the SE and NW Elysium chemical provinces^6^,^7^ and crater counting techniques to estimate crustal ages for each area. ArcGIS software in conjunction with the Mars Orbiter Laser Altimeter (MOLA) data was used to highlight the areas of interest. These highlighted areas were saved as shape files. Within each area, craters were marked by defining three points around the rim, before being subsequently exported to the CraterStats2 program. The program then generated a crater diameter vs cumulative crater frequency graph in order to calculate the estimated ages for the two provinces^41^.

In the present work, the data from all γ pixels in the martian mid-latitudes, other than those identified as the two Elysium Chemical Provinces, collectively represent the bulk composition of the martian crust, down to depths of several decimeters^29^. Each GRS pixel has a spatial resolution of several hundred km^2^. The small fractional area of Mars occupied by the Elysium chemical provinces also ensures that other data pixels approximate the bulk crust characterized before by Taylor *et al*.^14^ and McSween *et al*.^51^. Specifically, while the extent of the SE and NW Elysium chemical provinces (Fig. 1) are about 1.37 × 10^6^ km^2^ and 5.06 × 10^5^ km^2^, respectively, they constitute a sufficiently small area to ensure that their exclusion from the bulk crustal values does not make the bulk crustal estimate unreliable. Their exclusion enables unambiguous insight into compositional trends distinct from those in the typical martian crust. For our investigations using the γ data, we use the maps constructed from cumulative gamma spectra mapping periods extending through 2009, refining the older compositional data for greater accuracy and precision that those used in Karunatillake *et al*.^2^. We also include previously unreported chemical maps for the elements Al and S. Our chemical data exclude latitudes poleward of ~±45°, where high H concentrations cause both mass dilution effects and inaccuracies in γ spectrum-to-elemental mass fraction derivation^2^,^11^. We use mid-to-low latitudinal mass fraction maps generated from the GRS instrument. We bin each of the maps from their original dimensions to 5° × 5° pixel size. This serves to reduce spatial uncertainty associated with the reduced degrees of freedom from spatial autocorrelation^52^,^53^. The radioactive elements K and Th are the two elements with the finest γ spatial resolution, owing to the orbiting instrument’s detection of natural radioactive decay and independence from neutron-driven scatter and capture processes, unique among the majority of elements detected by the GRS instrument^11^. It is for this reason that K and Th are the primary elements utilized for interpretation of geologic processes.

In the ratio bound box plot method, the ratio of the 25th percentile of a particular element’s reported mass fraction within a region “A” to the 75^th^ percentile of values for the same element within a region “B” would bound how low the values are within “A” relative to those present within “B”. The ratio of the 25^th^%tile of “A”/75^th^%tile of “B” is represented by the bottom of each “box” in Fig. 4. Likewise, the ratio of the 75th percentile of region “A” compared to the 25^th^ percentile within “B” bounds how high the values within “A” can be relative to those within “B”. The ratio of the 75^th^%tile of “A”/25^th^%tile of “B” is represented by the top of each “box” in Fig. 4. The ratio of the 50^th^ percentile of “A” to the 50th percentile of region “B” (ratio of medians) indicates how the typical compositions compare with one another^53^, represented in the mid-section of each “box”. The error bars were propagated for the ratio of the 50^th^%tile/50^th^%tile using the median absolute deviations (MAD) for each element in both regions, using the equation MAD = median [|X_i_-median (X_i_)|]^54^, where median (X_i_) is the median of all values in X_i_. These error bars are statistically robust and typically overcompensate the uncertainty of the values of various %tiles. When constructing the ratio bound box plot in Fig. 4B, the normalization to a volatile-free basis was done on a pixel-by-pixel. This prevented the normalization from being effectively canceled out as a common denominator in the ratios.

The method for MgO abundance calculation used in this study uses oxide ratios reported from martian meteorites to calculate values for the following oxides: Na_2_O (Na_2_O/TiO_2_ = 1.374 ± 0.337), TiO_2_ (TiO_2_/P_2_O_5_ = 1.151 ± 0.265), P_2_O_5_ (P_2_O_5_/K_2_O = 4.297 ± 0.931), and MnO (MnO/FeO = 0.025 ± 0.001)^40^. Their calculated values were added to the oxide values converted from the elemental γ spectral data (Al_2_O_3_, CaO, FeO, SiO_2_, and K_2_O) and normalized to be volatile-free. The subsequent value was subtracted from 100, and the remaining value was assumed to represent the MgO content, as Mg is the only remaining major rock forming element. Major caveats exist in this calculation and uncertainties are very large. To partially address these concerns, we ignore any GRS pixels for which these calculations return Mg# < 40 or >70, as it is not consistent with an igneous crust from an initial mantle composition of roughly Mg# 75 28,40. The Mg# was calculated by using the mole fraction of the oxides MgO and FeO with the formula [Mg# = 100 * (MgO/(MgO + FeO))].

The oxide data used in the construction of the geochemical ternary plot for the martian meteorites were obtained by previous studies in laboratory settings. They were accessed online from the publically available Martian Meteorite Compendium (http://curator.jsc.nasa.gov/antmet/mmc/). Similarly, the *in situ* samples analyzed with the Alpha Particle X-ray Spectrometer (APXS) by the MER rovers (Spirit) at Gusev Crater were obtained online, publically available at MERAnalyst, (https://an.rsl.wustl.edu/), part of NASA’s Planetary Data Systems interface.

## Additional Information

**How to cite this article:** Susko, D. *et al*. A record of igneous evolution in Elysium, a major martian volcanic province. *Sci. Rep.* **7**, 43177; doi: 10.1038/srep43177 (2017).

**Publisher's note:** Springer Nature remains neutral with regard to jurisdictional claims in published maps and institutional affiliations.
